# Hydration of magnesia cubes: a helium ion microscopy study

**DOI:** 10.3762/bjnano.7.28

**Published:** 2016-02-29

**Authors:** Ruth Schwaiger, Johannes Schneider, Gilles R Bourret, Oliver Diwald

**Affiliations:** 1 Institute for Applied Materials (IAM) and Karlsruhe Nano Micro Facility (KNMF), Karlsruhe Institute of Technology (KIT), Hermann-von-Helmholtz-Platz 1, D-76344 Eggenstein-Leopoldshafen, Germany; 2 Department of Chemistry and Physics of Materials, University of Salzburg, Hellbrunnerstrasse 34/ III, A-5020 Salzburg, Austria

**Keywords:** helium ion microscopy, magnesia nanocubes, nanomaterials aging, oxide nanomaterials, surface hydroxylation, thin water films, volume expansion

## Abstract

Physisorbed water originating from exposure to the ambient can have a strong impact on the structure and chemistry of oxide nanomaterials. The effect can be particularly pronounced when these oxides are in physical contact with a solid substrate such as the ones used for immobilization to perform electron or ion microscopy imaging. We used helium ion microscopy (HIM) and investigated morphological changes of vapor-phase-grown MgO cubes after vacuum annealing and pressing into foils of soft and high purity indium. The indium foils were either used as obtained or, for reference, subjected to vacuum drying. After four days of storage in the vacuum chamber of the microscope and at a base pressure of *p* < 10^−7^ mbar, we observed on these cubic particles the attack of residual physisorbed water molecules from the indium substrate. As a result, thin magnesium hydroxide layers spontaneously grew, giving rise to characteristic volume expansion effects, which depended on the size of the particles. Rounding of the originally sharp cube edges leads to a significant loss of the morphological definition specific to the MgO cubes. Comparison of different regions within one sample before and after exposure to liquid water reveals different transformation processes, such as the formation of Mg(OH)_2_ shells that act as diffusion barriers for MgO dissolution or the evolution of brucite nanosheets organized in characteristic flower-like microstructures. The findings underline the significant metastability of nanomaterials under both ambient and high-vacuum conditions and show the dramatic effect of ubiquitous water films during storage and characterization of oxide nanomaterials.

## Introduction

Knowledge about the stability of engineered nanomaterials in aqueous systems is critical for predicting their functionality under environmental conditions. For example, for transient electronics it has become a major challenge to understand and control the factors of materials transformation in aqueous systems [1–2]. Under ambient conditions, nanostructured and highly dispersed metal oxides are instantaneously coated with thin water films. These films provide an unexplored reaction medium with an essentially unknown interface chemistry affecting both the structural and functional properties of oxide materials under operational conditions [3–4].

Among the many microscopic techniques available [5–6] scanning electron microscopy (SEM) and helium ion microscopy (HIM) [7] have been extensively employed in the fields of materials science [8–9]. Over the last years, HIM has developed into a high-performance alternative to the SEM. HIM is well-known for superior edge resolution reaching the low sub-nanometer range in secondary electron (SE) imaging [10] and excellent performance on insulating and biological samples [11–14]. Its extreme surface sensitivity in SE-based images [15] makes it very well suited for imaging of surface and interface details. Mostly deeper sample areas are affected by ion-induced damage and therefore the SE images are not directly compromised [16]. In addition to the high SE-yield, helium ion microscopy allows the use of low beam currents for imaging [7]. For biological materials and polymers, HIM is preferable to SEM for high resolution imaging due to the problems related to electron-beam-induced damage [17]. However, shrinkage of PMMA after helium ion imaging at 30 kV was reported [18], showing that also helium ions may damage soft materials by radiolysis just like low-energy (below 1 keV) electron beams. Thus, without additional damage to soft materials, HIM facilitates high resolution imaging, most importantly, without coating the samples for charge compensation. Sample charging effects that typically occur during imaging of insulating samples can be counteracted in the HIM by using a low-energy electron flood gun for charge compensation [7]. Although charging is a problem for MgO, in this study the flood gun was not needed because a metallic substrate was used.

Additionally, metal oxides can also be damaged by an electron beam [17]. Indeed, imaging metastable oxide and hydroxide nano- and mesostructures with SEM is difficult due to the effect the electron beam may have on the sample. For example, the drilling of holes into MgO smoke crystals by an electron beam was reported and different surface processes were suggested to occur [19]. Electron-beam irradiation can generate defects and potential nucleation sites for different transformation processes [20], and can thus induce the growth of thin solids such as lamellar hydroxides or hydroxide shells on top of the metal oxide cubic particles [21]. Because of its high resolution and surface sensitivity, HIM is particularly interesting for the characterization of reactive nanomaterials such as MgO, for which the unintended contact to residual water may become critical. For such systems, the influence of sample substrates on the chemical stability of the specimen needs to be investigated in detail.

The density mismatch that follows the conversion from MgO to Mg(OH)_2_ (from ρ_magnesia_ = 3.5 g·cm^−3^ to ρ_brucite_ = 2.3–2.4 g·cm^−3^) generates a significant volume expansion. For refractory materials, related phenomena can have desired [22] as well as undesired consequences for the macroscopic materials properties [23]. In the present study we employed this characteristic effect to trace the presence of spurious amounts of adsorbed water on the stability of MgO cubes that were chemically activated by vacuum annealing prior to the microscopy experiment.

There is growing awareness in the scientific community [24] that a variety of inconsistencies in nanomaterials properties reported in the literature originate from differing synthesis, processing, and environmental conditions that are experienced by particles in the different studies. This study describes the stability and transformation behavior of cubic metal oxide structures after their exposure to water in gaseous and liquid form. The influence of contact with water on the stability of morphologically well-defined MgO particle systems was investigated. We observed unexpected transformations ranging from swelling of the metal oxide nanostructure core to dissolution–recrystallization steps that give rise to the formation of hydroxides with entirely different microstructures [21].

## Experimental

All images were recorded using a high vacuum Orion Plus helium ion microscope (HIM, Carl Zeiss Microscopy GmbH, Germany). The microscope is equipped with an Everhardt–Thornley (ET) detector to record secondary electron images. The acceleration voltage of the ions and the beam current were 30 kV and approx. 0.25 pA, respectively. The MgO cube powders were vacuum annealed at *T* = 1173 K for a minimum duration of one hour in order to eliminate surface adsorbates including residual hydroxyls of high thermal stability [25]. After breaking the vacuum, we immobilized the particles in air by pressing the powder with a spatula into a high purity indium foil of 130 μm thickness (purity 99.99%, Alfa Aesar, 12206 LOT: N17A040). This soft and conductive substrate was used as-received or dried through vacuum treatment (base pressure below 3·10^−7^ mbar) at room temperature for four days. The latter procedure was applied in order to remove physisorbed species such as weakly bound water that forms during exposure of the foil to the ambient. The shape change of the cubes was quantified by image analysis, using the edge length increase factor, defined as *L*_2_/*L*_1_, where *L*_1_ and *L*_2_ are the edge lengths before and after high-vacuum treatment in the HIM chamber, respectively. Since it is not possible to simultaneously measure the three edge lengths of the cubes, we calculated the corresponding volume increase factor, defined as (*L*_2_/*L*_1_)^3^. More than 45 edge lengths were measured before and after high-vacuum treatment, for both types of samples (i.e., when the indium foil was used as received or subjected to vacuum drying prior to imaging). The cubes selected for the measurements had an estimated maximum tilt angle of 30° relative to the incident ion beam. This does not change the edge length increase factor (*L*_2_/*L*_1_), but induces a maximum error of less than 15% in edge length (*L*_1_). To verify that the tilt did not, indeed, affect our measurements, we measured cubes that were lying exactly flat on the surface (tilt = 0°) and found essentially the same dependence of *L*_2_/*L*_1_ on *L*_1_ (Supporting Information File 1, Figure S1). After analyzing the vacuum-treated sample, it was exposed to de-ionized water for 17 h and then again investigated in the HIM to evaluate the morphological changes.

## Results and Discussion

### Reactive water attack on MgO cubes under high vacuum (*p* < 3·10^−7^ mbar)

**Figure 1:**
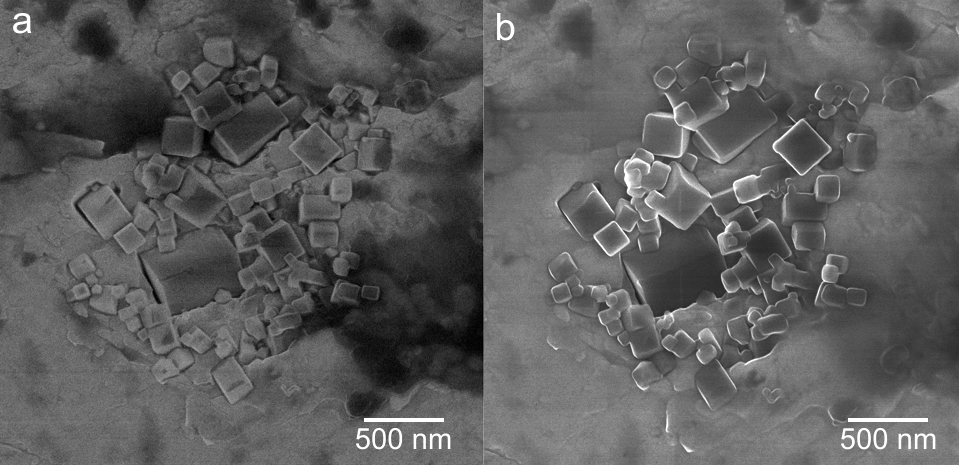
Effect of environmental water under high-vacuum conditions. Low-magnification HIM images of MgO cubes embedded in an indium foil after (a) 1 h and (b) 4 days in the HIM chamber (chamber pressure *p* < 3·10^−7^ mbar). Rounding of the MgO cube corners and edges is clearly visible in (b). (HIM SE images recorded at 30 kV acceleration voltage, (a) 0.3 pA beam current, 2.5·10^14^ cm^−2^ ion dose and (b) 25 kV acceleration voltage, beam current 0.1 pA, 3.0·10^14^ cm^−2^ ion dose).

Figure 1 shows MgO cubes being partially embedded in the untreated indium foil right after the mounting procedure and after four days in the HIM vacuum chamber, respectively.

**Figure 2:**
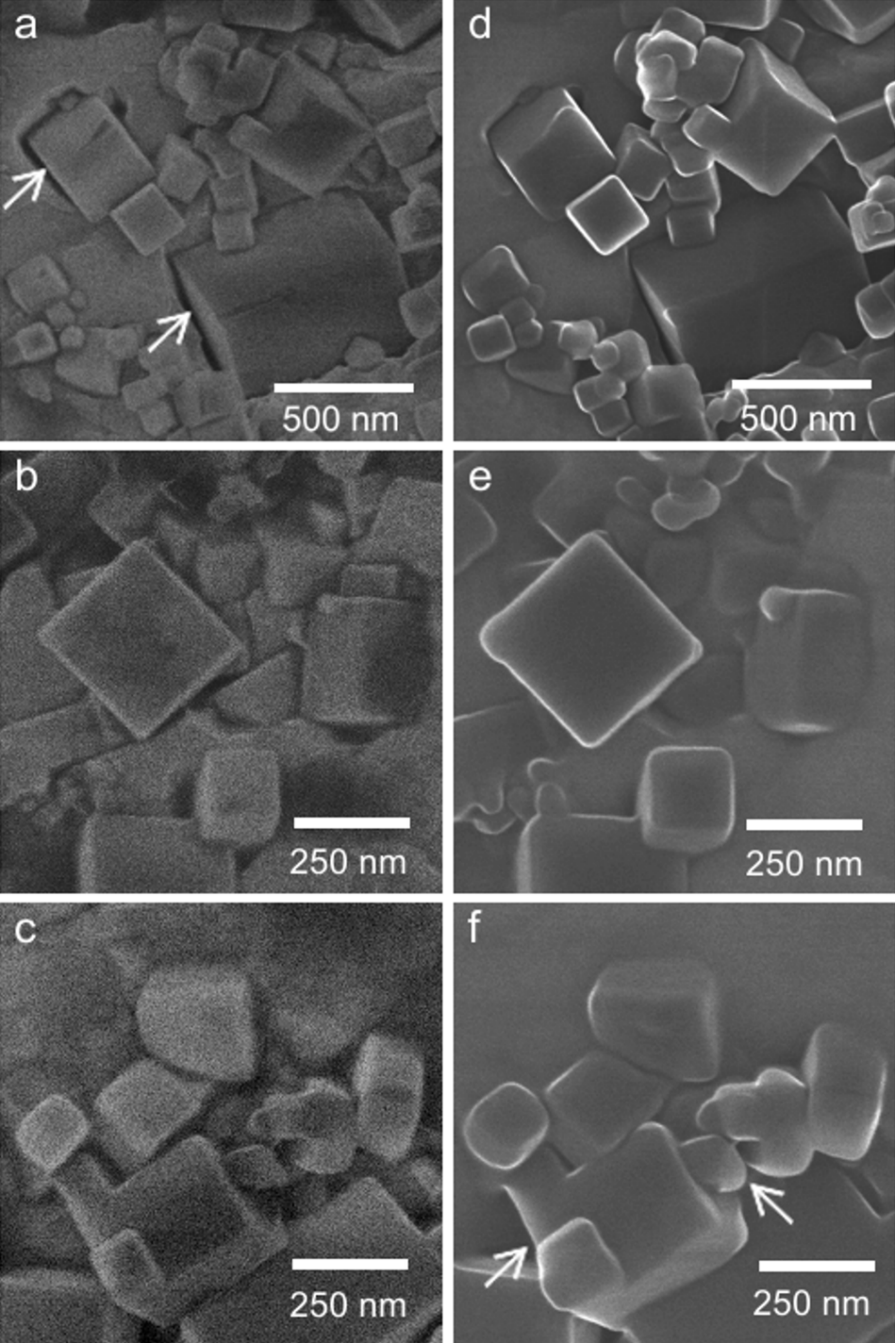
Morphological changes of MgO cubes under high-vacuum conditions. (a–c) High-magnification HIM images of MgO cubes embedded in an indium foil after 1 hour and (d–f) 4 days in the HIM chamber (chamber pressure *p* < 3·10^−7^ mbar). Significant volume increase, rounding of the edges, and partial fusion of the particles was observed (see arrows in micrographs). (HIM SE images digitally magnified from Figure 1.)

After four days under high-vacuum conditions at a base pressure lower than 3·10^−7^ mbar, HIM imaging reveals that important morphological changes of the MgO cubes have occurred (Figure 1). As shown in greater detail in Figure 2, the originally sharp corner and edge features appear rounded, which is attributed to the preferential erosion of more reactive corner and edge features [21]. This leads to a significant loss of morphological particle definition. Moreover, the MgO cubes have expanded presumably due to the formation of MgO/Mg(OH)_2_ core–shell structures, and a thick Mg(OH)_2_ layer coats the MgO cubes, leading to a partial fusion of the particles.

**Figure 3:**
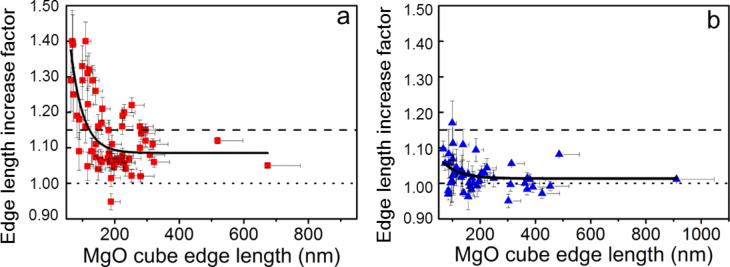
Quantification of morphological changes: Curves showing the edge length increase factor of MgO cubes (*L*_2_/*L*_1_) as a function of the edge length *L*_1_, where *L*_1_ and *L*_2_ are the edge lengths before and after storage in the vacuum chamber of the HIM, respectively. (a) The indium foil was used without any further treatment. The dashed line corresponds to a full transformation of MgO into Mg(OH)_2_ (i.e., *L*_2_/*L*_1_ = 1.15). The dotted line corresponds to zero expansion (*L*_2_/*L*_1_ = 1). The *y*-error bars correspond to the uncertainty caused by the HIM measurement (pixel element size related to magnification and image resolution). The *x*-error bars correspond to the possible effect of the tilt (maximum 30° tilt, inducing a possible underestimation of the edge length of 15%). The continuous black lines (exponential fits) are shown as guides to the eye. (b) The indium foil was dried under vacuum before being used as a substrate.

As shown in Figure 3, the expansion of the MgO cubes is significant. We measured an average edge length increase factor *L*_2_/*L*_1_ of 1.14 ± 0.10 (standard deviation of the measurement) when the indium foil was used as received, where *L*_1_ and *L*_2_ are the edge lengths before and after high vacuum treatment, respectively. This corresponds to a volume increase factor (*L*_2_/*L*_1_)^3^ of 1.52 ± 0.44 (for details concerning the measurement, see the Experimental section). In the case of a complete transformation of MgO cubes into Mg(OH)_2_ cubes, a similar volume increase factor, i.e., 1.52, would be expected. The high value of the volume expansion measured is attributed to the Kirkendall effect, which has been observed many times in the case of solid-to-solid transformations [26–28]. Such transformations can very often lead to hollow structures due to the depletion of the reactant in the center of the nanoparticle that diffuses to react on the outside of the nanoparticle. In such cases, the volume expansion after the reaction can significantly exceed the theoretical value of a complete solid-to-solid transformation. Differences in diffusion coefficient between the reacting species (here Mg^2+^, and H_2_O molecules/hydroxide ions) are thus expected to lead to the growth of the Mg(OH)_2_ layer outwards the cubes, explaining the large increase in volume measured here. The proposed transformation process, which is schematically shown in Figure S2 of Supporting Information File 1, involves physisorbed water. This water originates from the contact of the high-purity indium foil with air and forms a thin water film in which the MgO cubes are immersed. As a reference, we measured the change in edge length of the cubes on the vacuum-dried foil and found a moderate average volume increase factor of 1.08 ± 0.14 after high-vacuum treatment.

Furthermore, a clear size dependence is observed as shown in Figure 3. Above approx. 250 nm edge lengths, the volume increase factor is consistently below 1.5 (i.e., edge-length increase factor ≈ 1.15), while a very large expansion can be measured for cubes with edge lengths smaller than 140 nm. For such small cubes, the volume increase factor can go up to about 2.5 (i.e., edge length increase factor ≈ 1.35). Of course, the accuracy of the HIM measurement decreases for smaller structures. However, the trend is quite clear. Such observations are in line with previous reports [21] showing that small MgO cubes are subject to faster dissolution as compared to larger ones.

**Figure 4:**
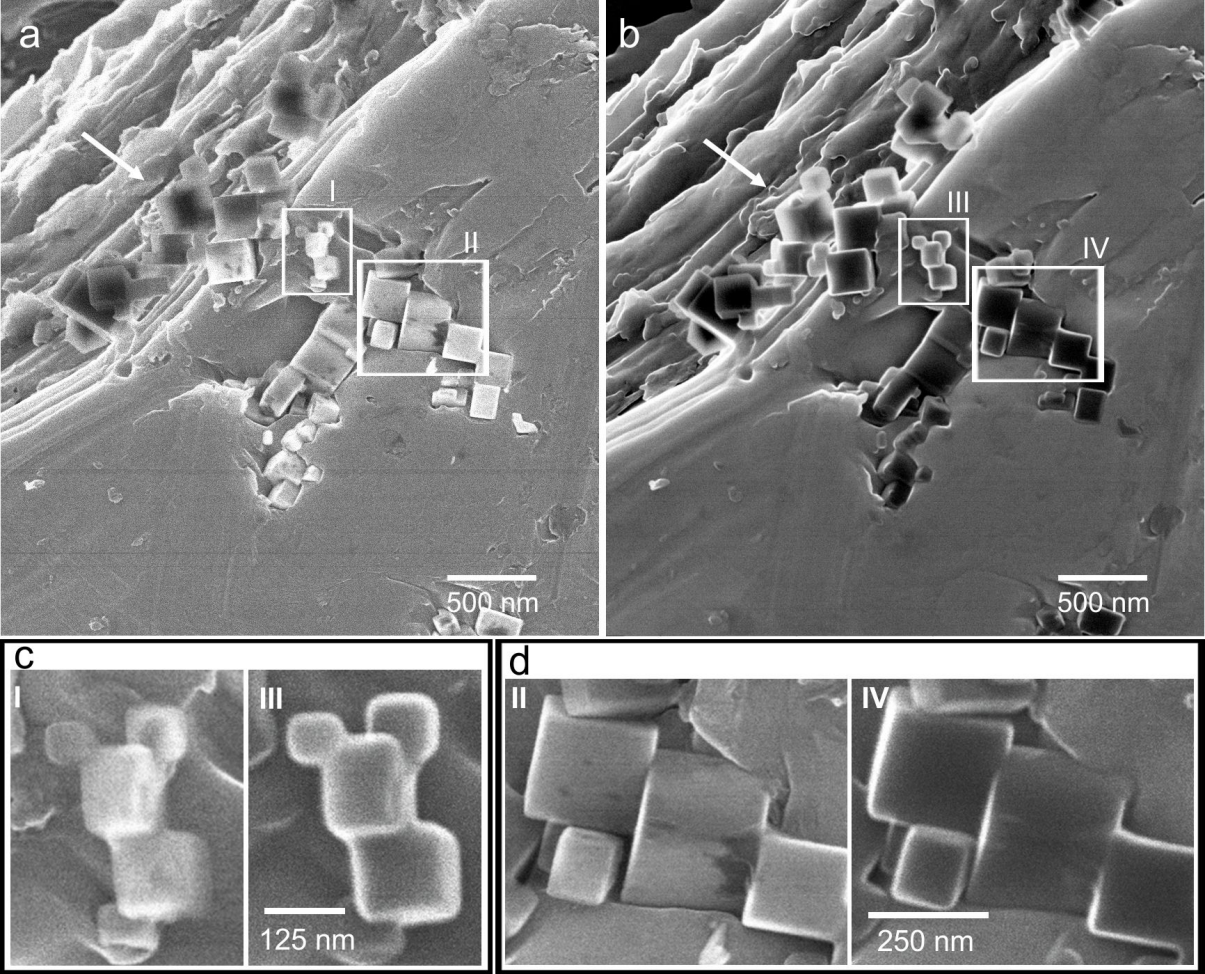
In addition to morphological changes under high-vacuum conditions, the MgO cubes embedded in the as-received indium foil after (a) 1 h and (b) 4 days in the HIM chamber (chamber pressure *p* < 3·10^−7^ mbar) exhibit contrast changes, which are attributed to the surface chemistry. MgO cubes that are not in contact with the indium foil (marked by arrows) do not exhibit any changes. (c,d) MgO cubes in areas I–IV at higher magnification illustrating both morphological and contrast changes. (HIM SE images recorded at 30 kV acceleration voltage and (a,b) 0.3 pA beam current, 5·10^14^ cm^−2^ ion dose, (c,d) 0.3 pA beam current, 2·10^15^ cm^−2^ ion dose.)

Interestingly, contrast changes between the different imaging sessions are quite evident (Figure 4). In this Figure, MgO cubes were imaged at a different location before and after four days of vacuum treatment. While the free-standing cubes exhibit comparable contrast and sharp edges (arrow in a) and b)), the ones in contact with the indium foil show rounded edges similar as in Figure 1 and Figure 2. The contrast of the cubes in contact with the foil and imaged after the four day period is quite different. Although we took great care in sample positioning, slight variations of working distance and specimen tilt could not be avoided. In addition, fluctuations in the beam current may have occurred. However, the contrast changes observed may also be indicative of chemical modifications; since SEs in the HIM are generated almost exclusively from the primary ion beam, they carry information about the surface chemistry [29].

The build-up of carbon on the sample areas during imaging may appear similar. As illustrated in [30], the secondary electron yield may decrease as a function of the ion dose due to the formation of carbon residues on the surface. However, in our case this effect is not predominant. First, cubes that were not in contact with the substrate did not show any swelling after the four day period (see arrows in Figure 4), and second, no morphological changes were observed when the cubes were pressed into the vacuum-annealed indium foil (Figure S3, Supporting Information File 1) and imaged with comparable total ion dose. The accumulated dose will at some point induce an unacceptable number of defects and a crystalline material will become amorphous [16]. It needs to be noted, though, because of the high ionicity of MgO its amorphization by ion implantation is difficult [31]. In this study, not more than five images per area were recorded, ensuring that the accumulated ion dose was below 5·10^15^ cm^−2^. Because the defects mainly occur close to the stopping range of the ions, which is ca. 160 nm according to the SRIM software [32], it is commonly considered to be an acceptable dose, because the material above the defects and the surface are unaffected [16].

**Figure 5:**
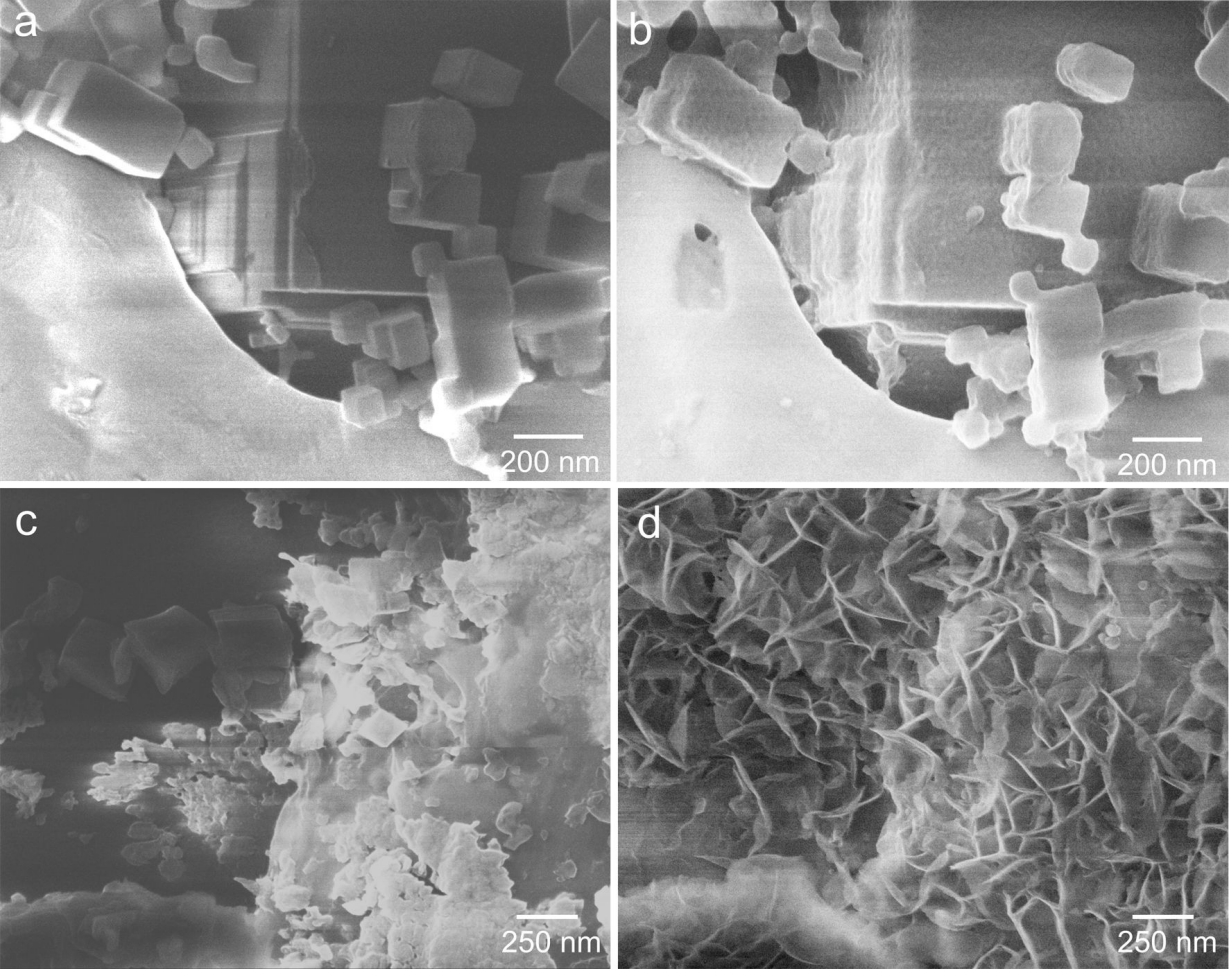
Helium ion microscopy images of vapor-phase-grown MgO cubes before (a,c) and after (b,d) exposure to liquid water. The sample areas imaged in (a) and (c) are the same as the ones imaged in (b) and (d), respectively. Clearly, different types of nanostructures can result from the interaction of MgO particle surfaces with H_2_O depending on their size and distribution at the sample surface. Comparison between (a) and (b) points to the volume expansion during hydration and hydroxylation, while (c) and (d) show a probable dissolution/precipitation mechanism. (HIM SE images recorded at 30 kV acceleration voltage and (a,c) 0.3 pA beam current, 1.4·10^15^ cm^−2^ ion dose, (b,d) 0.1 pA beam current, 1.2·10^15^ cm^−2^ ion dose.)

We performed exploratory experiments to address the impact of liquid water on thermally activated MgO cubes. A comparison was performed on sample spots having cubes of different sizes put in contact with a water droplet (Figure S4, Supporting Information File 1). The sample position characterized by the image in Figure 5 shows larger MgO cubes before (Figure 5a) and after 17 h of exposure to liquid water (Figure 5b). In that case the material exhibits the morphological changes discussed above, i.e., the evolution of a conformal hydroxide layer on top of the cubes paralleled by volume expansion. Moreover, originally sharper surface features such as cube corners and edges have become rounded in the course of this treatment. Previous studies on size-dependent dissolution effects [21] have revealed that hydroxide film formation limits mass transfer and, thus, may hamper the further dissolution process through the surface hydroxide [33]. This is consistent with the observations made on the larger cubes. An entirely different situation, however, can be found for smaller cubes that are characterized by the materials changes shown in Figure 5c,d. In that case microstructures composed of flower brucite sheets have emerged from the significant dissolution of smaller MgO cubes, the size of which does not prevent them from further dissolution [21]. As a result, the aqueous solution becomes locally supersaturated in Mg^2+^ and OH^−^ ions and lamellar Mg(OH)_2_ structures crystallize and give rise to flower-like morphologies (Figure 5d) [21,34–35]. In addition to nanostructured brucite Mg(OH)_2_, such crystal morphologies have been reported for very different materials systems such as barite (BaSO_4_) [36], as a mineralogical text-book system, or crystalline organic polymers such as polyimides that were grown by hydrothermal crystallization [37–38]. Independent of the chemical composition, nucleation is initiated at screw dislocations. Further crystallization proceeds non classically due to a strong growth anisotropy and different attachment energies for the monomer units at particular positions on the growth layer [36,39]. The two cases of materials transformation (Figure 5), which were observed on a powder sample covered by a water droplet, clearly reveal entirely different local reaction scenarios in the absence of convective mixing. A more detailed study of these functional dependences is underway.

## Conclusion

This work underlines the metastability of metal oxide nanostructures and reports drastic material changes during storage under high vacuum conditions. We observed significant and size-dependent volume expansion effects of the MgO cubes that were grown and processed in dry vacuum environments prior to imaging with helium ion microscopy. The volume expansions observed are attributed to surface reactions between the MgO cubes and water molecules originating as physisorbed species from an indium foil that was employed as a conductive substrate for imaging. Depending on their size the MgO-based cubes [21] become subject to significant volume expansion effects (up to a factor of 2.5) that are attributed to oxide transformation into hydroxides and the generation MgO/Mg(OH)_2_ core–shell structures as a result of the Kirkendall effect.

These results are significant and should be relevant for everyone working with nanomaterials under ambient and high-vacuum conditions. Conductive substrates require appropriate drying and surface cleaning prior to sample immobilization and measurement. Apart from the transformation behavior of cubic oxides such as MgO, NiO and CdO and its relevance for the synthesis of hydroxides with controlled morphologies [40–41], the phenomena reported herein underline the difficulties of maintaining desired nanomaterials properties during handling and processing due to their dynamic nature [24].

## Supporting Information

File 1 Additional experimental data.

## References

[1] Li R, Cheng H, Su Y, Hwang S-W, Yin L, Tao H, Brenckle M A, Kim D-H, Omenetto F G, Rogers J A (2013). Adv Funct Mater.

[2] Hwang S-W, Park G, Cheng H, Song J-K, Kang S-K, Yin L, Kim J-H, Omenetto F G, Huang Y, Lee K-M (2014). Adv Mater.

[3] Hwang S-W, Park G, Edwards C, Corbin E A, Kang S-K, Cheng H, Song J-K, Kim J-H, Yu S, Ng J (2014). ACS Nano.

[4] Gheisi A, Sternig A, Redhammer G J, Diwald O (2015). RSC Adv.

[5] König T, Simon G H, Heinke L, Lichtenstein L, Heyde M (2011). Beilstein J Nanotechnol.

[6] Su D S, Zhang B, Schlögl R (2015). Chem Rev.

[7] Hlawacek G, Veligura V, van Gastel R, Poelsema B (2014). J Vac Sci Technol, B.

[8] Zavyalova U, Geske M, Horn R, Weinberg G, Frandsen W, Schuster M, Schlögl R (2011). ChemCatChem.

[9] Vollnhals F, Drost M, Tu F, Carrasco E, Späth A, Fink R H, Steinrück H-P, Marbach H (2014). Beilstein J Nanotechnol.

[10] Hill R, Faridur Rahman F H M (2011). Nucl Instrum Methods Phys Res, Sect A.

[11] Bazou D, Behan G, Reid C, Boland J J, Zhang H Z (2011). J Microsc.

[12] Boden S A, Asadollahbaik A, Rutt H N, Bagnall D M (2012). Scanning.

[13] Joens M S, Huynh C, Kasuboski J M, Ferranti D, Sigal Y J, Zeitvogel F, Obst M, Burkhardt C J, Curran K P, Chalasani S H (2013). Sci Rep.

[14] Campo E M, Larios E, Huynh C, Ananth M (2015). J Mater Res.

[15] Castaldo V, Withagen J, Hagen C, Kruit P, van Veldhoven E (2011). Microsc Microanal.

[16] Livengood R, Tan S, Greenzweig Y, Notte J, McVey S (2009). J Vac Sci Technol, B.

[17] Egerton R F, Li P, Malac M (2004). Micron.

[18] Joy D C (2011). AIP Conf Proc.

[19] Turner P S, Bullough T J, Devenish R W, Maher D M, Humphreys C J (1990). Philos Mag Lett.

[20] Marbach H (2014). Appl Phys A.

[21] Baumann S O, Schneider J, Sternig A, Thomele D, Stankic S, Berger T, Grönbeck H, Diwald O (2015). Langmuir.

[22] Salomão R, Arruda C C, Souza A D V, Fernandes L (2014). Ceram Int.

[23] Salomão R, Souza A D, Fernandes L, Arruda C C (2013). Am Ceram Soc Bull.

[24] Baer D R, Engelhard M H, Johnson G E, Laskin J, Lai J, Mueller K, Munusamy P, Thevuthasan S, Wang H, Washton N (2013). J Vac Sci Technol, A.

[25] Diwald O, Sterrer M, Knözinger E (2002). Phys Chem Chem Phys.

[26] Yin Y, Rioux R M, Erdonmez C K, Hughes S, Somorjai G A, Alivisatos A P (2004). Science.

[27] Cabot A, Ibáñez M, Guardia P, Alivisatos A P (2009). J Am Chem Soc.

[28] Bourret G R, Goulet P J G, Lennox R B (2011). Chem Mater.

[29] Ward B W, Notte J A, Economou N P (2006). J Vac Sci Technol, B.

[30] Hlawacek G, Veligura V, Lorbek S, Mocking T F, George A, van Gastel R, Zandvliet H J W, Poelsema B (2012). Beilstein J Nanotechnol.

[31] Burnett P J, Page T F (1986). Radiat Eff Defects Solids.

[32] Ziegler J F, Ziegler M D, Biersack J P (2010). Nucl Instrum Methods Phys Res, Sect B.

[33] Ringleb F, Sterrer M, Freund H-J B (2014). Appl Catal, A.

[34] Das P S, Dey A, Mandal A K, Dey N, Mukhopadhyay A K (2013). J Adv Ceram.

[35] Gao Y, Wang H, Su Y, Shen Q, Wang D (2008). J Cryst Growth.

[36] Benning L G, Waychunas G A, Brantley S L, Kubicki J D, White A F (2008). Nucleation, Growth, and Aggregation of Mineral Phases: Mechanisms and Kinetic Controls. Kinetics of Water-Rock Interaction.

[37] Unterlass M M (2015). Mater Today.

[38] Baumgartner B, Puchberger M, Unterlass M M (2015). Polym Chem.

[39] Pina C M, Becker U, Risthaus P, Bosbach D, Putnis A (1998). Nature.

[40] Zhang S, Zeng H C (2009). Chem Mater.

[41] Shukla A K, Ercius P, Gautam A R S, Cabana J, Dahmen U (2014). Cryst Growth Des.

