# Supplementary material for: Hydration of magnesia cubes: a helium ion microscopy study
Source: Beilstein J Nanotechnol. 2016 Feb 29;7:302–9. doi: 10.3762/bjnano.7.28 (PMC4901542; doi:10.3762/bjnano.7.28)
Supplement: File 1 — Additional experimental data. [file Beilstein_J_Nanotechnol-07-302-s001.pdf]

Supporting Information

for

Hydration of magnesia cubes: a helium ion microscopy study

Ruth Schwaiger*[1], Johannes Schneider[2], Gilles R. Bourret[2] and Oliver Diwald*[2]

Address: [1]Institute for Applied Materials (IAM) and Karlsruhe Nano Micro Facility (KNMF), Karlsruhe Institute of Technology (KIT), Hermann-von-Helmholtz-Platz 1, D-76344 Eggenstein-Leopoldshafen, Germany and [2]Department of Chemistry and Physics of Materials, University of Salzburg, Hellbrunnerstrasse 34/ III, A-5020 Salzburg, Austria

Email: Ruth Schwaiger* - ruth.schwaiger@kit.edu;

Oliver Diwald* - oliver.diwald@sbg.ac.at

* Corresponding author

Additional experimental data

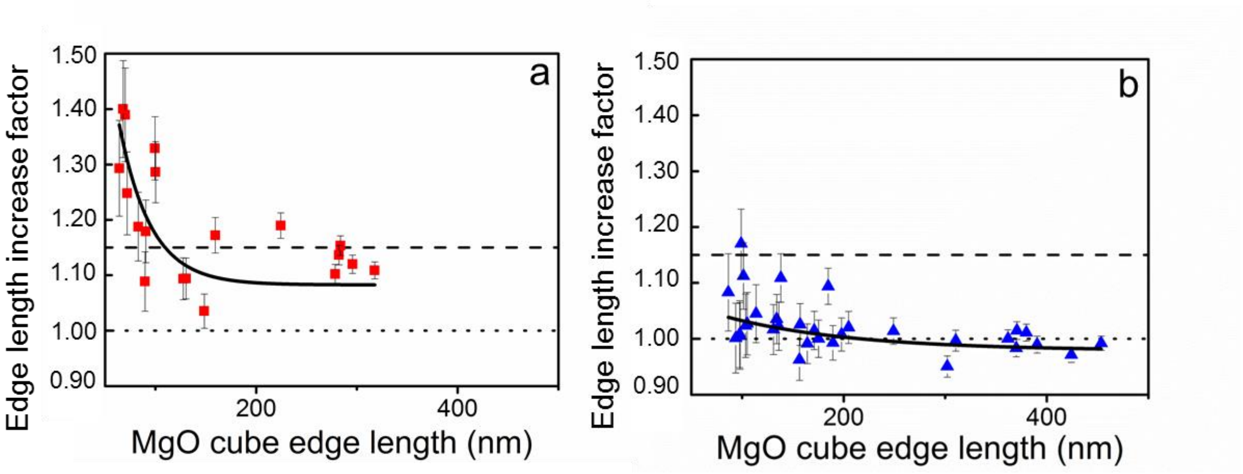

Figure S1: Effect of the tilt on the quantification of morphological changes. Only cubes that were exactly lying flat on the indium foil were measured (i.e., the incident ion beam was normal to the cube surface). (a,b) Curves showing the edge length increase factor of MgO cubes (L_2/L_1) as a function of edge length L_1, where L_1 and L_2 are the edge lengths before and after high vacuum treatment, respectively. (a) The indium foil was used without any further treatment. The dashed line corresponds to a full transformation of MgO into $Mg(OH)_2$ (i.e., $L_2/L_1 = 1.15$). The dotted line corresponds to zero expansion ($L_2/L_1 = 1$). The errors bars correspond to the uncertainty caused by the HIM measurement (pixel element size related to magnification and image resolution). The continuous black lines (exponential fits) are shown as guides to the eye. (b) The In foil was dried under vacuum before being used as a substrate.

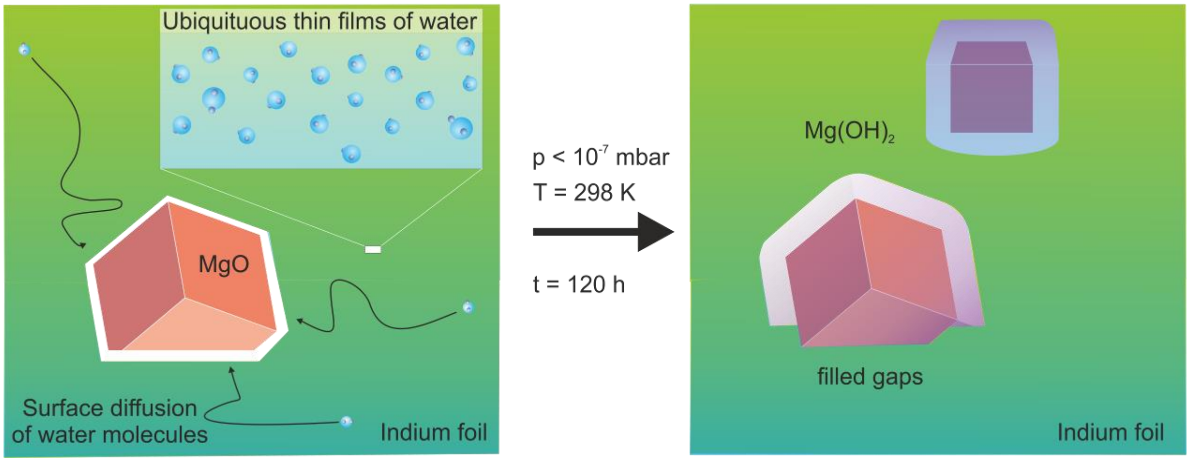

Figure S2: Proposed mechanism of superficial H_2O induced transformation of MgO cubes into MgO with $Mg(OH)_2$ coatings. This reaction step is associated with characteristic volume expansion effects that are consistent with the observations described above.

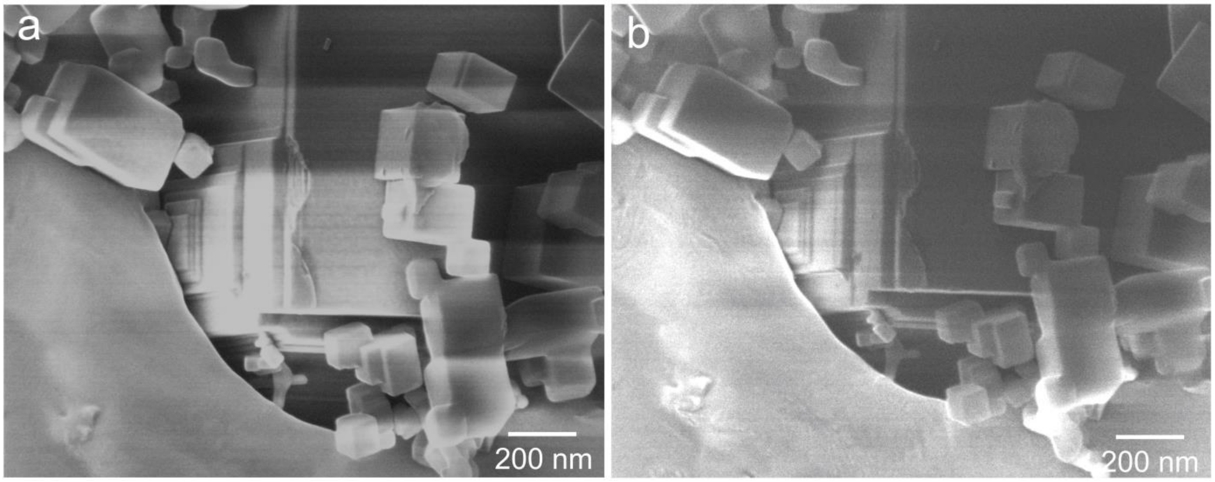

Figure S3: Helium ion microscopy of MgO nanocubes embedded in a vacuum-dried indium foil. Before pressing the MgO cubes into the foil, it was vacuum-dried under high vacuum conditions for four days. (a) MgO cubes after sample mounting, and (b) after 4 days under high vacuum conditions in the HIM chamber ($p < 3 \cdot 10^{-7}$ mbar).

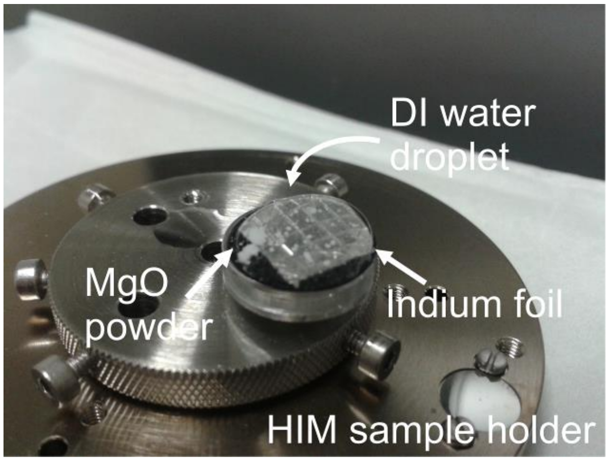

Figure S4: Digital photograph of the water droplet covering the particle powder, parts of which were imaged by HIM (see Figure 5 of main manuscript).
